# Low fibrosis biomarker levels predict cardiac resynchronization therapy response

**DOI:** 10.1038/s41598-019-42468-4

**Published:** 2019-04-15

**Authors:** Grégoire Massoullié, Vincent Sapin, Sylvain Ploux, Patrick Rossignol, Aurélien Mulliez, Frédéric Jean, Pierre-Yves Marie, Charles Merlin, Bruno Pereira, Marius Andronache, Pascal Motreff, Xavier Chabin, Jean-Marc Sellal, Bernard Citron, Jean-René Lusson, Charles Vorilhon, Guillaume Clerfond, Pierre Bordachar, Faiez Zannad, Romain Eschalier

**Affiliations:** 1 Cardiology Department, CHU Clermont-Ferrand, Clermont-Ferrand, France; 2 Clermont Université, ISIT-CaVITI, Clermont-Ferrand, France; 3 Department of Biochemistry, CHU Clermont-Ferrand, Clermont-Ferrand, France; 4 Hôpital Cardiologique du Haut-Lévêque, CHU Bordeaux, Université Bordeaux, IHU LIRYC, Bordeaux, France; 5 INSERM, UMR-1116; Université de Lorraine; INSERM CIC 1433, Nancy, France; 6 INI-CRCT F-CRIN, Nancy, France; 7 Biostatistics Unit (Clinical Research and Innovation Direction), CHU Clermont-Ferrand, Clermont-Ferrand, France; 8 INSERM, UMR-1116; Université de Lorraine; CHRU-Nancy, Service de Médecine Nucléaire, Nancy, France; 9 Nuclear Medicine, Centre Jean Perrin, Clermont-Ferrand, France; 10 Cardiology Department, CHRU-Nancy, Nancy, France

## Abstract

Cardiac fibrosis is associated with heart failure and poor prognosis. Fibrosis biomarkers have been poorly evaluated as a tool to predict cardiac resynchronization therapy (CRT) response generating conflicting results. The present study assessed the predictive value of cardiac fibrosis biomarkers on CRT response. Patients underwent clinical examination, echocardiography and blood fibrosis biomarker evaluation prior to CRT implantation. At six months, a positive response to CRT was defined by a composite endpoint of no death or hospitalization for heart failure, and presence of left ventricular (LV) reverse remodeling (decrease in LV end-systolic volume ≥15%). Sixty patients were included in a multicenter study. At 6 months, 38 were positive responders to CRT and reached the response criteria (63%). Compared to non-responders, CRT responders displayed lower concentration levels of the fibrosis biomarkers procollagen type I C-terminal propeptide [PICP 135[99–166] ng/ml *vs*. 179[142–226]ng/ml, p = 0.001)] and procollagen type III N-terminal propeptide [PIIINP 5.50[3.66–8.96] ng/ml vs. 8.01[5.01–11.86]ng/ml, p = 0.014)] at baseline. In multivariate analysis, a PICP ≤ 163 ng/ml was associated with a positive CRT response [OR = 7.8(1.3–46.7), p = 0.023] independently of the presence of LBBB, QRS duration, LV lead position or non-ischemic cardiomyopathy. Altogether, the present findings show that a lower degree of cardiac fibrosis is associated with a positive response after CRT implantation. PICP evaluation before CRT implantation could help improve patient selection.

## Introduction

Cardiac resynchronization (CRT) is an effective treatment in addition to optimal drug therapy for heart failure with reduced ejection fraction (HFrEF)^1–3^. Initial research on resynchronization therapy was purely clinical, enabling the establishment of recommendations for the implantation of CRT based on the presence of symptomatic heart failure (NYHA II to ambulatory IV) with left ventricular dysfunction (LVEF ≤ 35%) and electrical dyssynchrony (QRS duration ≥130 ms and QRS morphology)^4,5^.

Unfortunately, the mechanisms underlying the response to CRT are poorly understood. As a result, 20 to 40% of patients implanted with CRT are non-responders irrespective of the endpoint chosen to assess CRT response^3,6^. Myocardial fibrosis has been identified as a determinant of the prognosis of cardiomyopathy progression^7,8^ as well as an indicator of a poorer prognosis of HFrEF^9,10^.

Extracellular matrix turnover, measured by circulating collagen peptides, is associated with ventricular remodeling in the case of hypertension after myocardial infarction and to excess mortality in the context of HF^11,12^. These biomarkers have been only sparsely studied in the setting of CRT^13,14^ and we hypothesize that lower levels of collagen peptides are likely associated with a better CRT response.

The aim of this first-known dedicated prospective study was to assess the contribution of the quantification of myocardial fibrosis by means of serological determination of collagen peptides prior to implantation in predicting the response to CRT.

## Material and Methods

Written consent for this prospective multicenter cohort study was obtained from each patient after receiving the appropriate information. PREFAC-CRT (NCT02018029) obtained the ethical approval from the French Ethics Committee and the French National Agency for Medicines and Health Products Safety and was conducted in accordance with European Society of Cardiology (ESC) guidelines^4,5^.

### Inclusion criteria

Patients were included according to the following criteria: age ≥18 years; indication for CRT according to the ESC 2012 guidelines; expected life expectancy greater than 1 year with good functional status; optimal pharmacological treatment of HF^5^. Non-inclusion criteria were: pregnancy; contraindication to CRT; atrial fibrillation; non-affiliation to a social security scheme; disability.

### Primary endpoint

Patients responding to CRT were defined at 6 months post-implantation by absence of hospitalization for HF or cardiovascular death defined by terminal heart failure death (obtained upon evaluation of the hospitalization report if the patient was not managed in our center) or sudden cardiac death related to ventricular arrhythmia documented on device monitoring and by a decreased indexed left ventricular end-systolic volume (LVESVi) of more than 15% (ΔLVESVi ≥ 15%) measured by 2D echocardiography.

### Initial examination

Medical workup at inclusion, prior to CRT implantation, included a clinical examination, resting transthoracic echocardiography, 6-minute walking test, quality of life score (Minnesota Living With Heart Failure Questionnaire: MLWHFQ), as well as a blood sample in conjunction with a serum biobank of cardiac fibrosis biomarkers. Ischemic heart disease was defined as a LV systolic dysfunction related to a previous cardiac event (myocardial infarction, coronary artery bypass graft). Non-ischemic dilated cardiomyopathy (DCM) was defined as a reduced LVEF not attributable to an ischemic, valvular or pacemaker-induced cardiomyopathy.

### Fibrosis biomarkers

ELISA kits (Cloud-Clone Corporation^®^, Katy, TX, USA) were used for determination of serum collagen peptide concentrations, namely: (i) biomarkers of collagen synthesis: PINP (Procollagen type I N-terminal propeptide), PICP (Procollagen type I C-terminal propeptide) and PIIINP (Procollagen type III N-terminal propeptide); and (ii) a biomarker of collagen degradation: ICTP (Collagen type I C-terminal telopeptide)^15,16^. Each patient sample was measured in duplicate, following the recommendations of the manufacturer. For the four determinations, intra-assay and inter-assay precision was <10% and <12%, respectively.

### Transthoracic echocardiography

2-D transthoracic echocardiography was performed using a 2.5 to 5 MHz imaging probe connected to a Vivid 9 ultrasonic device (Vingmed-General Electric, Horten Norway) in accordance with the recommendations of the American Society of Echocardiography and the European Association of Cardiovascular Imaging^17^. Image analysis was subsequently performed by two blinded operators.

### CRT implantation and device programming

In each investigating center, implantation and programming methods were left to the discretion of each operator with the goal of the highest rate of biventricular pacing. Information regarding venous anatomy (number and position of the coronary veins), the position of the leads as well as programming parameters was collected. Post-procedural complications were also duly noted.

### Statistical analysis

Sample size estimation was determined in order to highlight an effect-size equal to 0.5 for a two-sided type I error at 0.01 (correction due to multiple tests) and a statistical power greater than 90%. With an expected rate of responders ranging between 50% and 70%, it was initially proposed to include 300 patients. However, further exploratory sequential analyses were performed to evaluate effect-size and estimate statistical power. As a result, with an effect-size of 0.89 [0.34; 1.44], a total of 60 patients allowed achieving a statistical power greater than 80%.

Statistical analyses were performed using Stata (version 12, StataCorp, College Station, US). All statistical tests were two-sided and a p-value < 5% was considered statistically significant. Continuous data are expressed as mean ± standard-deviation or median [interquartile range], according to statistical distribution (assumption of normality assessed using the Shapiro-Wilk test). Comparisons among CRT responses were performed using the chi-squared or Fisher’s exact tests for categorical data, and using Student’s t-test or Mann-Whitney test (when assumptions of the t-test were not met) for continuous data. Hedge’s g bias-corrected effect size and its 95% confidence interval were computed and plotted for each biomarker. To analyze relationships between biomarkers, remodeling and LVESVi, Pearson or Spearman’s correlation coefficients were computed, applying a Sidak’s correction to take into account multiple comparisons.

A multivariable analysis (i.e. generalized linear model with logit link function) was thereafter performed to study the predictive factors of CRT response. The covariates were determined according to univariate results and clinical relevance. Particular attention was given to the study of multicollinearity and interactions between covariates 1) studying the relationships between the covariables and 2) assessing the impact of adding or deleting variables in the multivariable model. Following this multivariable analysis, a receiver operating characteristic (ROC) curve was plotted for the final model, along with computation of the area under the curve (AUC) and its 95% confidence interval. In order to assess the contribution of PICP to CRT-response prediction, two multivariable models were generated: one with LBBB, dilated cardiomyopathy, QRS duration (without PICP) and lateral LV lead position and the second using the same covariates plus PICP. The models were thereafter compared using the Likelihood-ratio test (LR test), the Akaike Information Criterion (AIC) and the Bayesian information criterion (BIC).

Since bootstrap validation does not require distributional assumptions (such as normally distributed errors), the latter can provide more accurate inferences when the data are not well behaved or when the sample size is small^18^. Thus, a bootstrap procedure^19,20^ was performed from the original sample, from which the bootstrap estimates associated with each covariate and their associated standard errors were averaged from replicates. Results are shown as adjusted odds-ratios and their 95% confidence interval.

Finally, to take into account potential confounders in the analysis of the effect of PICP on CRT response (due to imbalance/differences in dilated etiology, LBBB and QRS duration between the 2 PICP groups), a sensitivity analysis was carried out, using a propensity score analysis with logistic regression model, to assess the propensity for patients to be in the PICP < 163 group, in which dilated etiology, LBBB and QRS duration were considered as covariates. The relationship between CRT response and PICP was subsequently analyzed using inverse propensity score weighting. Results are shown as odds-ratios and 95% confidence interval.

## Results

### Study population

**Figure 1:**
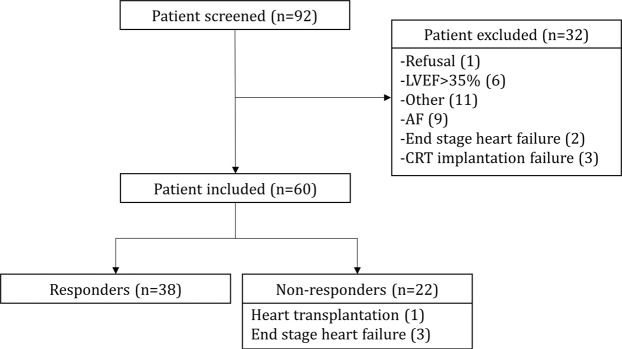
Study flow chart.

Sixty patients were included and successfully implanted (80% CRT-D). Early repositioning of the left ventricular lead was necessary in one patient while another was treated for early device infection. For the latter two patients, follow-up was only initiated once their CRT system was fully functional. At 6 months, 56 patients underwent transthoracic echocardiography; of the remaining 4 patients, 3 died from terminal cardiac failure while one patient underwent a heart transplant. At 6 months, 38 patients (63%) were responders to CRT (Fig. 1).

### Responders vs. Non-responders

NYHA improvement at 6 months was 0.27 ± 0.78 *vs*. 0.79 ± 0.87, p = 0.04, QRS duration at 6 months was 152 ± 39 ms *vs*. 147 ± 26 ms, p = 0.57, QRS thinning was 8 ± 37 ms *vs*. 25 ± 30 ms, p = 0.005 and 6-meter walking distance improvement was 8 ± 93 *vs*. 1 ± 152, p = 0.6 in non-responders and responders, respectively (Table 1).

**Table 1:** Characteristics at baseline.

	Total (n = 60)	Responders (n = 38)	Non-responders (n = 22)	p value
Age(year)	69 ± 10	68 ± 10	69 ± 10	0.820
Female sex	15%(9)	18%(7)	9%,(2)	0.464
BMI(kg/m²)	28 ± 5	27 ± 5	29 ± 5	0.281
Hypertension	55%(33)	58%(22)	50%(11)	0.561
Dyslipidemia	62%(37)	55%(21)	73%(16)	0.186
Obesity	32%(19)	29%(11)	36%(8)	0.560
Diabetes mellitus	28%(17)	21%(8)	41%(9)	0.103
History of Heart Failure	55%(33)	50%(19)	64%(14)	0.314
Chronic Respiratory Insufficiency	15%(9)	5%(2)	32%(7)	0.006
Denutrition	3%(2)	NA	9%(2)	0.060
Non-ischemic cardiomyopathy	42%(25)	55%(21)	18%(4)	0.005
IHM	45%(27)	29%(11)	73%(16)	0.001
Heart Rate(bpm)	68 ± 11	66 ± 10	71 ± 13	0.140
NYHA	2.41 ± 0.65	2.16 ± 0.68	2.25 ± 0.59	0.153
6MWT(m)	365 ± 167	385 ± 187	326 ± 116	0.216
MLWHFQ	32 ± 18	29 ± 18	39 ± 15	0.031
QRS duration(ms)	163 ± 24	172 ± 22	147 ± 19	< 0.001
LBBB	52%(31)	63%(24)	32%(7)	0.019
NICD	27%(16)	13%(5)	50%(11)	0.002
RBBB	5%(3)	3%(1)	9%(2)	0.276
Paced	17%(10)	21%(8)	9%(2)	0.238
Estimated GFR(/ml/min/1.73 m²)	69.1 ± 24.6	71.8 ± 24.5	64.5 ± 24.8	0.272
NT-ProBNP(ng/dl)	3547 ± 8492	2753 ± 4558	4919 ± 12764	0.345
Heart Failure Drugs				
Beta-blockers	80%(47)	81%(30)	77%(17)	0.750
ACEi/ARBs	82%(49)	84%(32)	77%(17)	0.512
MRA	37%(22)	34%(13)	41%(9)	0.600
Ivabradine	17%(10)	16%(6)	18%(4)	0.849
Loop diuretics	80%(48)	76%(29)	86%(19)	0.510
Echocardiography				
Indexed end-diastolic volume(ml/m²)	99 ± 25	91 ± 17	104 ± 28	0.049
Indexed end-systolic volume(ml/m²)	71 ± 21	64 ± 17	75 ± 22	0.041
LVEF(%)	28 ± 6	29 ± 7	28 ± 6	0.660
PAPs(mmHg)	34 ± 12	28 ± 9	40 ± 13	0.003
LV Lateral lead position	82%(49)	89%(34)	68%(15)	0.040
CRT-D	80%(48)	79%(30)	82%(18)	0.118

6MWT = Six-minute walking test; ACEi = Angiotensin-converting enzyme inhibitor; ARBs = angiotensin receptor blockers; BMI = Body mass index;; eGFR = estimated glomerular filtration rate; IHM = Ischemic heart disease; LBBB = Left bundle branch block; LVEF = left ventricular ejection fraction; MLWHFQ = Minnesota Living With Heart Failure Questionnaire; MRA = Mineralocorticoid antagonist receptor; NICD = Non-specific intraventricular conduction delay; NYHA = New York Heart Association; PAPs = Pulmonary arterial pressure; RBBB = Right bundle branch block.

Patients who responded to CRT more frequently had, prior to CRT implantation, a LBBB (63% *vs*. 32%, p = 0.019), DCM (55% *vs*. 18%, p = 0.005), wider QRS duration (172 ± 22 ms *vs*. 147 ± 19 ms, p < 0.001), a better quality of life (i.e. lower MLWHFQ: 29 ± 18 *vs*. 39 ± 15, p = 0.031) and a LV lead positioned in a lateral vein of the coronary sinus (89% *vs*. 68%, p = 0.040). Patients not responding to CRT more often had respiratory insufficiency (32% *vs*. 5%, p = 0.006) and required positive inotropic amines in the month preceding implantation (12.5% *vs*. 0%, p = 0.031).

### Cardiac fibrosis biomarkers

**Figure 2:**
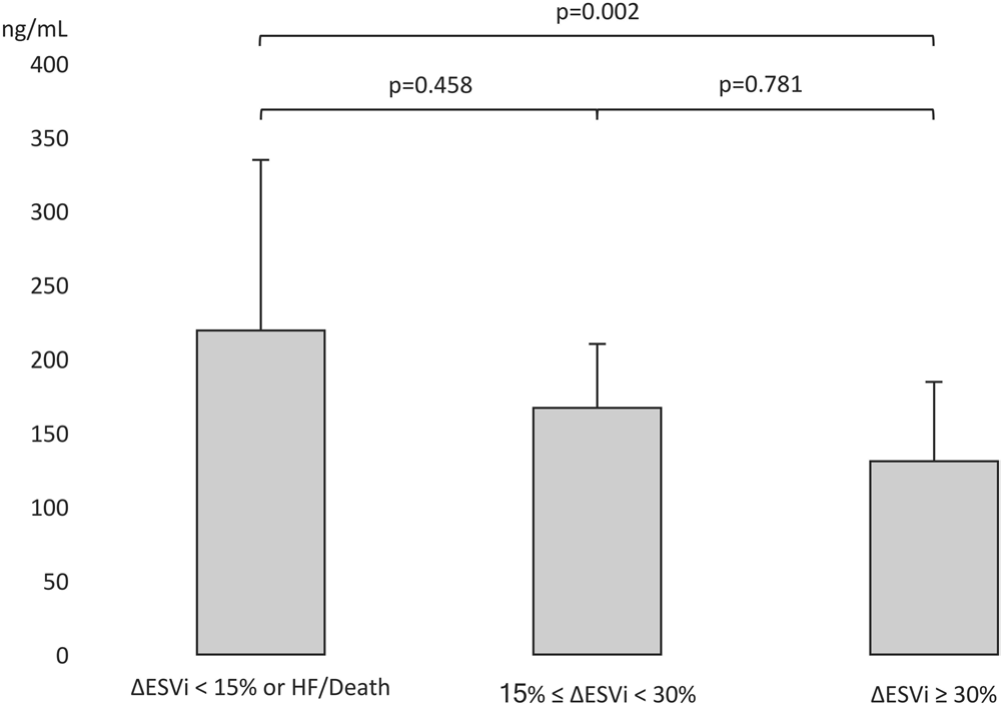
PICP level according to CRT response and left ventricular remodeling at 6 months. ΔESVi: Indexed End-systolic Volume variation at 6 months; HF: heart failure.

CRT responders had lower PICP (135 [99–166] *vs*. 179 [142–226] ng/ml, p = 0.001) and PIIINP (5.50 [3.66–8.96] *vs*. 8.01 [5.01–11.86] ng/ml, p = 0.014) levels than non-responders. Patients with a higher inverse remodeling (ΔLVESVi ≥ 30%) had a lower PICP than non-responder patients (Fig. 2). PICP was inversely correlated with left ventricular remodeling at 6 months (r = −0.468, p < 0.001) and with end-systolic volume at inclusion (r = −0.354, p = 0.004). PICP level was weakly correlated with PIIINP (r = −0.271, p = 0.036) (Table 2).

**Table 2:** Serum collagen peptide levels at baseline among responders and non-responders to CRT.

	Total (n = 60)	Responders (n = 38)	Non-responders (n = 22)	p value
PICP(ng/mL)	148[112–200]	135[99–166]	179[142–226]	0.001
ICTP(ng/mL)	1.09[0.82–1.40]	1.04[0.81–1.36]	1.16[0.83–1.71]	0.865
PINP(ng/mL)	3.85[3.39–4.53]	3.83[3.39–4.63]	3.93[3.38–4.5]	0.835
PIIINP(ng/mL)	6.65[4.24–9.94]	5.50[3.66–8.96]	8.01[5.01–10.86]	0,014
PICP/ICTP	126.5[92.9–215.9]	116.9[78.2–195.2]	158.7[106.1–323.8]	0.049
PIIINP/ICTP	58.5[39.5–108.5]	52.5[31–89]	92.5[44–118]	0.036

ICTP = Collagen type I C-terminal telopeptide; PICP = Procollagen type I C-terminal propeptide; PIIINP = Procollagen type III N-terminal propeptide PINP = Procollagen type I N-terminal propeptide.

**Figure 3:**
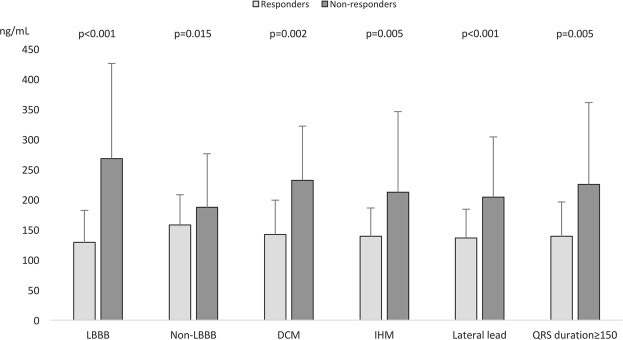
PICP level according to CRT response and baseline characteristics. ΔESVi: Indexed End-systolic Volume variation at 6 months. LBBB = Left bundle branch block; DCM = Non-ischemic cardiomyopathy; LV = Left ventricle.

Patients not responding to CRT in spite of recognized predictive factors of response had a higher PICP level: i.e. in the presence of a LBBB (268 ± 158 *vs*. 129 ± 53 ng/ml, p < 0.001), DCM (232 ± 90 *vs*. 142 ± 57 ng/ml, p = 0.015) or QRS duration ≥ 150 ms (225 ± 136 *vs*. 139 ± 57 ng/ml, p = 0.005) (Fig. 3). Lastly, PICP, PIIINP, PINP and ICTP levels did not statistically differ in patients treated with MRA or ACE/ARAII, irrespective of whether patients were responders or not.

Multivariable analysis with bootstrap procedure showed that PICP was an independent CRT response factor [OR = 7.8 (1.3–46.7)] (Table 3). A sensitivity analysis using inverse propensity score weighting confirmed these results.

**Table 3:** Multivariate analysis of factors associated with a CRT response.

	OR[95%CI]	p-value
LBBB*	8.9[1.4–58.7]	0.023
Non-ischemic cardiomyopathy	24.9[2.6–240.4]	0.005
PICP ≤ 163 (ng/mL)	7.8[1.3–46.7]	0.023
QRS duration ≥ 150 ms	28.2[3–263.9]	0.003
Lateral lead position	5.1[0.7–40.6]	0.120

LBBB = Left Bundle Branch Block (*QRS > 130 ms); PICP = Procollagen type I C-terminal propeptide.

**Figure 4:**
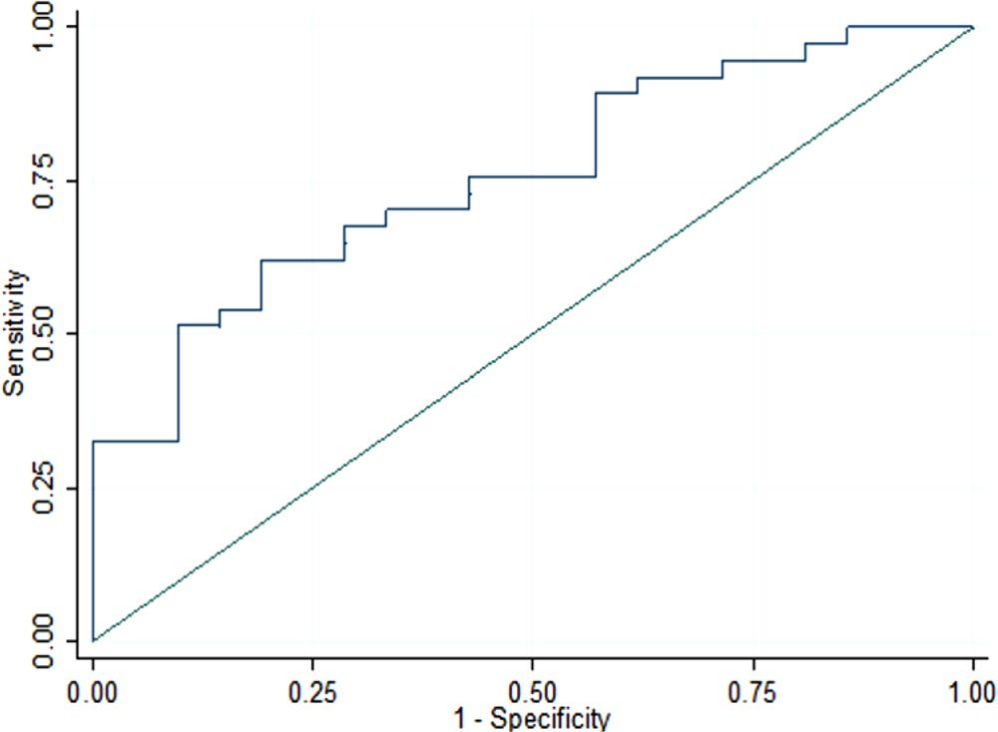
Response to CRT ROC curve according to PICP level (AUC = 0.75[0.62–0.87]).

PICP effect size (on CRT response) was 0.89 [0.34–1.44] (Table 4). PICP had an area under the ROC curve predicting a CRT response of 0.75 [0.62–0.87] in the whole study population (Fig. 4), and of 0.90 [0.73–1], 0.83 [0.60–1] and 0.78 [0.61–0.93] among patients with LBBB, DCM or QRS ≥ 150 ms. The contribution of PICP, in addition to LBBB, dilated cardiomyopathy and QRS duration, was statistically significant (LR test: p = 0.003), together with better AIC (50 with *vs*. 57 without PICP) and BIC (61 *vs*. 65).

**Table 4:**
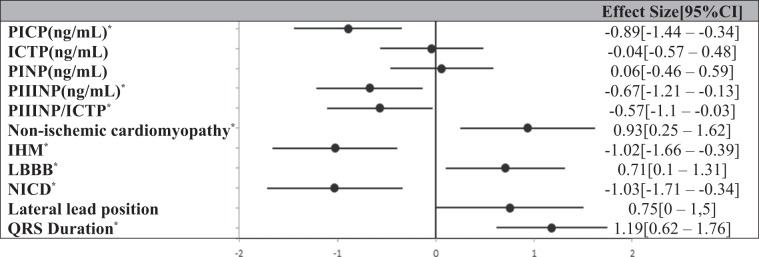
Size effect of collagen peptides according to CRT response at 6 months.

IHM = Ischemic heart disease; ICTP = Collagen type I C-terminal telopeptide; LBBB = Left bundle branch block; PICP = Procollagen type I C-terminal propeptide; PIIINP = Procollagen type III N-terminal propeptide; PINP = Procollagen type I N-terminal propeptide; NICD = Non-specific intraventricular conduction delay. ^*^Significant effect size on predictive value.

## Discussion

This novel prospective study enabled assessing the impact of myocardial fibrosis evaluated by circulating collagen peptides on the response to CRT. The findings obtained herein highlight several major elements in the understanding of the response to CRT that could not be previously explained by isolated analysis of electrical or mechanical dyssynchrony. These new elements notably include: 1) the response to CRT is associated with a lower PICP rate prior to implantation while CRT non-responders who are otherwise expected to respond (i.e. LBBB, DCM and QRS duration ≥150 ms^20^) display a higher PICP level; 2) left ventricular remodeling is inversely associated with the degree of biological fibrosis despite the presence of electrical dyssynchrony or non-ischemic heart disease; and 3) a low PICP level (≤163 ng/ml) is independently associated (LBBB, DCM and QRS duration) with a positive response to CRT; PICP thus adds a new value in predicting CRT response, in addition to the above classical typical parameters validated as CRT response predictors.

More than a decade of research has not allowed implementing a reliable additional criterion in the selection of CRT responders. We demonstrate herein that PICP ≤ 163 ng/ml was associated with a 7.8-fold increase in the likelihood of being a responder (p = 0.023) and was also correlated with LV remodeling at 6 months. This translates to a greater degree of myocardial fibrosis at implantation^12^ and a lower degree of reverse remodeling in response to CRT^21^. This interstitial fibrosis is currently not taken into account by the two traditional indications for CRT implantation: electrical dyssynchrony and left ventricular contractile function. CRT first and foremost corrects an electrical disease; however the response rate is not optimal, even in patients deemed to be high responders (i.e. LBBB, DCM, QRS duration ≥150 ms)^22^. The present study shows that despite the presence of typical response factors, these non-responder patients have a higher serum PICP. Patients with advanced heart diseases, with a significant degree of fibrotic remodeling, have a limited response to CRT^23^, partly explaining why these patients do not improve despite the presence of classical predictive factors. Thus, evaluation of PICP prior to CRT implantation would enable a better identification of patients prone to respond to CRT, including those expected as responders.

Focal fibrotic myocardial scars are identified using imaging techniques^23,24^. By limiting LV scar pacing and harboring aspecific conduction disorders, these scars ultimately reduce CRT response. MRI provides accurate localization of focal scars, although to a lesser extent with regard to quantification of the interstitial fibrotic infiltrate in the ventricular myocardium as a whole. Quantifying this infiltrative and diffusive fibrosis remains challenging, despite the advent of promising new imaging sequences^7,8^. Response to cardiac resynchronization is likely more dependent on the overall volume of interstitial fibrosis^23,25^. In this respect, collagen peptides and notably PICP are of prime interest, since they allow a quantitative assessment of the fibrotic burden of the myocardium, albeit with no localizing value. Non-invasive myocardial fibrosis quantification could therefore represent the missing link in the individual evaluation of patients with CRT.

Biomarker candidates for assessing cardiac fibrosis in clinical practice are rare^26,27^. Collagen peptides have been poorly used in the study of CRT response by determining the degree of myocardial fibrosis prior to implantation in addition to yielding conflicting results. Lopez-Andres *et al*. observed that increased Galectin-3 and PIIINP were associated with adverse long-term cardiovascular outcomes but did not predict the response to CRT^12^. These findings are inconsistent with a retrospective analysis of the MADIT-CRT trial, where Galectin-3 elevation was inversely correlated with heart failure and death after CRT^28^. Umar *et al*.^14^ also highlighted the potential impact of collagen peptides (PINP) to predict CRT response without direct comparison of PICP with PINP.

With regard to PICP, Garcia-Bolao *et al*. showed that collagen type I turnover (higher PICP/ICTP ratio in responders) influenced the long-term response to CRT (12 months), similarly to their previously published study^13^. Unfortunately, PICP was not independently evaluated from other response criteria. Surprisingly, they described a higher level of fibrosis associated with CRT responders in contrast with data provided by Umar *et al*.^14^, Lopez *et al*.^12^ as well as the present study in which patients with lower levels of fibrosis were prone to respond to CRT. The reason for this discrepancy is unknown, but may be related to variations in CRT indications as a result of guideline evolution, inconsistent CRT response criteria based either on left ventricle remodeling (LVESVi > 10% at 6 months^14^), functional status (6 minute walking test^13^), morbidity (major cardiovascular outcomes^28^) or a composite thereof (LVEF > 35% and survival or NT-ProBNP < 1000 pg/ml and survival^12^), somehow poorly correlated^29^, and lastly a lack association between histological myocardial fibrosis in human biopsies^30^ and fibrosis biomarkers such as Galectin-3 and PINP. The method of collagen peptide assessment does not appear to be a probative explanation since radioimmunoassay was used in the Umar *et al*., Lopez *et al*. and Garcia-Bolao *et al*. studies.

Collagen peptides are associated with the total volume of type I and III collagens constituting the majority of the extracellular matrix^31^ whose dysregulation in synthesis/degradation balance increases their accumulation. Clinically, the serum level of PICP is strongly associated with the total fraction of type I collagen in the myocardium^32^, particularly in instances of heart failure or diastolic dysfunction^33,34^. PICP and PIIINP are associated with the presence of myocardial fibrosis in tissue biopsies as opposed to ICTP or PINP or Galectin-3^27^. Our results confirm PICP as a promising fibrosis marker that is both minimally invasive and reproducible.

The biological evaluation of cardiac fibrosis is an attractive and credible alternative to standard imaging techniques. While minimally invasive, it would also allow improving patient management and contribute to the understanding of the mechanisms of HF and the response to CRT. The systematic measurement of PICP prior to CRT implantation, would optimize such management, especially those with a high PICP level. Identifying less responding patients could enable more aggressive management, whether medically or interventionally. Although we did not observe an association between the prescription of anti-fibrotic treatments (e.g. MRA) and collagen peptide levels, non-responder patients could benefit from an intensification of these molecules before and/or during CRT implantation. As demonstrated in post-acute myocardial infarction^35^, part of the myocardial fibrosis is reversible by anti-fibrotic treatments. CRT, as also the case for standalone cardiac electrical dyssynchrony therapy, does not appear to impact the rate of fibrosis^12,14^, albeit with conflicting results^13^ and thus requires further dedicated studies. Unfortunately, evaluation of collagen peptides after CRT implantation was not performed in the present study but would be of great interest especially with the impact of antifibrotic drugs.

In terms of study limitations, it should be emphasized that our study comprises a limited population sample. However, the validity of this cohort is confirmed by the identification of conventional CRT response criteria. In addition, despite the small sample size of this study, we nonetheless demonstrate the clinical value of PICP both at baseline and in multivariate analysis. More precisely, PICP effect size (0.89 [0.34; 1.44]) can be considered high and relevant, according to Cohen’s recommendations^36^.

In the present study, the biological evaluation of cardiac fibrosis was not compared with another technique. MRI sequences are used to quantify interstitial fibrosis which is correlated with the measurement of collagen peptides in the absence of heart failure. This non-invasive method was not widely disseminated at the start of the study and had poorly defined measurement standardization^25^. CRT non-responders had a lower rate of LV lead implantation in the lateral vein of the coronary sinus. A non-optimal position is associated with a higher non-response which could overwhelm the role of fibrosis although the former had a limited size effect in our population (Table 4). Finally, our CRT response endpoint was a clinical and echocardiographic composite. Evolution of the LVESVi is strongly associated with an improvement in survival and corresponds to a robust clinical assessment of the response^37^.

## Conclusion

Low serum levels of PICP are predictive factors independent of a positive CRT response at 6 months. This study demonstrates the relevance of the biological assessment of cardiac fibrosis prior to CRT implantation in addition to the traditional strongly validated predictive factors (i.e. LBBB, DCM and QRS duration ≥ 150 ms).

## Data Availability

The datasets generated and analyzed during this study are included in the published article and are also available from the corresponding author upon request.

## References

[1] Cazeau S (2001). Effects of multisite biventricular pacing in patients with heart failure and intraventricular conduction delay. N. Engl. J. Med..

[2] Cleland JGF (2005). The effect of cardiac resynchronization on morbidity and mortality in heart failure. N. Engl. J. Med..

[3] Abraham WT (2002). Cardiac resynchronization in chronic heart failure. N. Engl. J. Med..

[4] Ponikowski P (2016). ESC Guidelines for the diagnosis and treatment of acute and chronic heart failureThe Task Force for the diagnosis and treatment of acute and chronic heart failure of the European Society of Cardiology (ESC)Developed with the special contribution of the Heart Failure Association (HFA) of the ESC. Eur. Heart J..

[5] Epstein AE (2013). 2012 ACCF/AHA/HRS focused update incorporated into the ACCF/AHA/HRS 2008 guidelines for device-based therapy of cardiac rhythm abnormalities: a report of the American College of Cardiology Foundation/American Heart Association Task Force on Practice Guidelines and the Heart Rhythm Society. Circulation.

[6] Bristow MR (2004). Cardiac-Resynchronization Therapy with or without an Implantable Defibrillator in Advanced Chronic Heart Failure. N. Engl. J. Med..

[7] Gulati A (2013). Association of Fibrosis With Mortality and Sudden Cardiac Death in Patients With Nonischemic Dilated Cardiomyopathy. JAMA.

[8] Halliday BP (2017). Association Between Midwall Late Gadolinium Enhancement and Sudden Cardiac Death in Patients With Dilated Cardiomyopathy and Mild and Moderate Left Ventricular Systolic Dysfunction. Circulation.

[9] Aoki T (2011). Prognostic impact of myocardial interstitial fibrosis in non-ischemic heart failure. -Comparison between preserved and reduced ejection fraction heart failure.-. Circ. J. Off. J. Jpn. Circ. Soc..

[10] Azevedo CF (2010). Prognostic significance of myocardial fibrosis quantification by histopathology and magnetic resonance imaging in patients with severe aortic valve disease. J. Am. Coll. Cardiol..

[11] Eschalier R (2013). Extracellular matrix turnover biomarkers predict long-term left ventricular remodeling after myocardial infarction: insights from the REVE-2 study. Circ. Heart Fail..

[12] Lopez-Andrès N (2012). Association of galectin-3 and fibrosis markers with long-term cardiovascular outcomes in patients with heart failure, left ventricular dysfunction, and dyssynchrony: insights from the CARE-HF (Cardiac Resynchronization in Heart Failure) trial. Eur. J. Heart Fail..

[13] García-Bolao I (2008). Impact of collagen type I turnover on the long-term response to cardiac resynchronization therapy. Eur. Heart J..

[14] Umar S (2008). Myocardial collagen metabolism in failing hearts before and during cardiac resynchronization therapy. Eur. J. Heart Fail..

[15] Fernández-Real JM (2012). A Mediterranean diet enriched with olive oil is associated with higher serum total osteocalcin levels in elderly men at high cardiovascular risk. J. Clin. Endocrinol. Metab..

[16] Flevari P (2012). Serum markers of deranged myocardial collagen turnover: their relation to malignant ventricular arrhythmias in cardioverter-defibrillator recipients with heart failure. Am. Heart J..

[17] Lang RM (2015). Recommendations for Cardiac Chamber Quantification by Echocardiography in Adults: An Update from the American Society of Echocardiography and the European Association of Cardiovascular Imaging. Eur. Heart J. - Cardiovasc. Imaging.

[18] Efron, B., Tibshirani, R., Davidson, A. & Hinkley, D. An introduction to the bootstrap, Bootstrap Methods and Their Applications. Cambridge Cambridge University Press (1997).

[19] Mooney, C. Z. & Duval, R. D. *Bootstrapping: A Nonparametric Approach to Statistical Inference*. (SAGE Publications, Inc, 1993).

[20] Goldenberg I (2011). Predictors of response to cardiac resynchronization therapy in the Multicenter Automatic Defibrillator Implantation Trial with Cardiac Resynchronization Therapy (MADIT-CRT). Circulation.

[21] Chen Z (2016). Focal But Not Diffuse Myocardial Fibrosis Burden Quantification Using Cardiac Magnetic Resonance Imaging Predicts Left Ventricular Reverse Modeling Following Cardiac Resynchronization Therapy. J. Cardiovasc. Electrophysiol..

[22] Cleland JG (2013). An individual patient meta-analysis of five randomized trials assessing the effects of cardiac resynchronization therapy on morbidity and mortality in patients with symptomatic heart failure. Eur. Heart J..

[23] Cochet H (2013). Pre- and intra-procedural predictors of reverse remodeling after cardiac resynchronization therapy: an MRI study. J. Cardiovasc. Electrophysiol..

[24] Khan FZ (2012). Targeted left ventricular lead placement to guide cardiac resynchronization therapy: the TARGET study: a randomized, controlled trial. J. Am. Coll. Cardiol..

[25] Leong DP (2012). Effects of myocardial fibrosis and ventricular dyssynchrony on response to therapy in new-presentation idiopathic dilated cardiomyopathy: insights from cardiovascular magnetic resonance and echocardiography. Eur. Heart J..

[26] Gyöngyösi M (2017). Myocardial fibrosis: biomedical research from bench to bedside. Eur. J. Heart Fail..

[27] López B (2015). Circulating Biomarkers of Myocardial Fibrosis: The Need for a Reappraisal. J. Am. Coll. Cardiol..

[28] Stolen CM, Adourian A, Meyer TE, Stein KM, Solomon SD (2014). Plasma galectin-3 and heart failure outcomes in MADIT-CRT (multicenter automatic defibrillator implantation trial with cardiac resynchronization therapy). J. Card. Fail..

[29] Fornwalt BK (2010). Agreement is poor among current criteria used to define response to cardiac resynchronization therapy. Circulation.

[30] Querejeta R (2000). Serum carboxy-terminal propeptide of procollagen type I is a marker of myocardial fibrosis in hypertensive heart disease. Circulation.

[31] Weber KT (1989). Cardiac interstitium in health and disease: the fibrillar collagen network. J. Am. Coll. Cardiol..

[32] Prockop DJ, Kivirikko KI (1995). Collagens: molecular biology, diseases, and potentials for therapy. Annu. Rev. Biochem..

[33] López B, Querejeta R, González A, Larman M, Díez J (2012). Collagen Cross-Linking But Not Collagen Amount Associates With Elevated Filling Pressures in Hypertensive Patients With Stage C Heart FailureNovelty and Significance. Hypertension.

[34] Querejeta R (2004). Increased Collagen Type I Synthesis in Patients With Heart Failure of Hypertensive Origin. Circulation.

[35] Iraqi W (2009). Extracellular Cardiac Matrix Biomarkers in Patients With Acute Myocardial Infarction Complicated by Left Ventricular Dysfunction and Heart Failure. Circulation.

[36] Cohen, J. *Statistical power analysis for the behavioral sciences (2nd ed*.*)*. *New Jersey: Lawrence Erlbaum*, *1988*.

[37] Yu C-M (2005). Left ventricular reverse remodeling but not clinical improvement predicts long-term survival after cardiac resynchronization therapy. Circulation.

